# Pituitary apoplexy: surgical or conservative? A meta-analytical insight

**DOI:** 10.3389/fsurg.2025.1579498

**Published:** 2025-06-04

**Authors:** Kailai Xia, Jihong Peng, Yunhao Zhou, Xiaoqing Liu, Hongyu Chen, Haiyang Xu, Shuangji Wang, Aini Deng, Hua Tang, Jinyang Hu

**Affiliations:** ^1^ Department of Neurosurgery, The First College of Clinical Medical Science, Three Gorges University & Yichang Central People’s Hospital, Yichang, Hubei, China; ^2^ College of Basic Medical Sciences, China Three Gorges University, Yichang, Hubei, China; ^3^ Department of Anesthesiology, The First College of Clinical Medical Science, Three Gorges University & Yichang Central People’s Hospital, Yichang, Hubei, China

**Keywords:** pituitary apoplexy, surgical treatment, conservative treatment, meta-analysis, systematic review

## Abstract

**Objectives:**

The challenge in treating pituitary apoplexy lies in choosing between conservative management and surgical intervention, with a current lack of high-level medical evidence to guide the selection of treatment options. This study compares the recovery rates of typical clinical manifestations following surgical and conservative treatments, aiming to provide evidence-based medical support for clinical treatment decisions.

**Methods:**

Relevant literature published between 1991 and 2024 was searched using PubMed, Embase, Cochrane Libraries, and CNKI. After a rigorous screening process to apply the inclusion and exclusion criteria, the primary clinical observation indicators were carefully extracted. The recovery rates of the main clinical observation indicators were evaluated using Reman v5.3. A fixed-effects model was employed to determine the merged odds ratio (OR) values, utilizing the Mantel-Haenszel estimation method. Publication bias was assessed using a funnel plot. Heterogeneity between studies was analyzed with the Cochran Q (Chi-square) test and I² statistics.

**Results:**

The meta-analysis results indicated that surgical treatment significantly improved recovery from ocular muscle paralysis compared to conservative treatment (OR: 0.31; 95% CI 0.10–0.92; *p* = 0.04). However, no statistically significant differences were observed in postoperative recovery of visual acuity (OR: 1.15; 95% CI 0.54–2.44; *p* = 0.72), visual field recovery (OR: 1.48; 95% CI 0.77–2.82; *p* = 0.24), or pituitary endocrine function (OR: 0.67; 95% CI 0.27–1.67; *p* = 0.38).

**Conclusion:**

Our research findings suggest that patients with pituitary apoplexy presenting with ocular palsy may benefit more from surgical treatment.

## Introduction

Pituitary apoplexy is a rare but serious condition resulting from acute hemorrhage or infarction of the pituitary gland, first described in 1898 (1). Patients with pituitary apoplexy commonly present with acute symptoms, including severe headaches, nausea, vomiting, and optic nerve dysfunction, which may be accompanied by ocular muscle paralysis. In severe cases, patients may also experience altered consciousness, posing a serious risk to life (2–4).

Upon confirming a diagnosis of pituitary apoplexy, it is crucial to develop an appropriate treatment plan tailored to the severity of the patient's clinical manifestations. This plan may encompass conservative measures, such as close monitoring and regular follow-up assessments, alongside neurosurgical interventions for more severe cases (5). However, there is still controversy over whether to choose surgical treatment or conservative therapy during the acute phase. In 2011, the United Kingdom and in 2013, Spain successively published the “Pituitary Apoplexy Management Guidelines” (6) and the “Clinical Practice Guidelines for the Diagnosis and Treatment of Pituitary Apoplexy (7),” aiming to provide clinical guidance and recommendations for the diagnosis and management of pituitary apoplexy, but there are shortcomings such as a lack of recommendation evidence and unclear diagnostic and treatment standards. In recent years, although China has also formulated a series of expert consensuses related to pituitary adenomas, there is still no unified standard for the diagnosis and treatment of pituitary apoplexy (8), and there is a lack of evidence-based medical evidence. European guidelines explicitly highlight that severe or progressive neurological deficits, such as acute vision loss, ophthalmoplegia, and consciousness disturbances, represent the primary indications for emergency surgery. In cases where patients exhibit only headache or mild visual impairment without progression, conservative management, including hormone replacement and close monitoring, is generally favored. Conversely, Chinese guidelines, such as those issued by the Chinese Medical Association, advocate for early surgical intervention in patients with moderate to severe visual impairment or significant mass effect evident on imaging studies. Additionally, some clinicians may adopt a more flexible approach, tailoring decisions based on the patient's endocrine status. The aim of our study is to investigate how various prognostic indicators influence the preference for surgical intervention and thereby provide guidance for clinical decision-making.

In this context, we analyzed nearly 30 years of global research data to evaluate the efficacy of surgical vs. conservative treatments for patients with pituitary apoplexy. This study aims to assess whether surgical intervention leads to superior outcomes compared to conservative treatment for pituitary apoplexy, based on recovery in visual and endocrine functions.

## Materials and methods

### Data analysis

We analyzed relevant literature published between 1991 and 2024 to conduct systematic reviews through a comprehensive search of several databases, including PubMed, Cochrane Library, and Web of Science. Our search utilized keywords and phrases like ‘pituitary apoplexy,’ ’surgical treatment,’ ‘conservative treatment,’ ‘prospective cohort studies,’ ‘randomized controlled trials,’ and ‘retrospective cohort studies,’ along with additional synonyms. As part of our search optimization approach, we used Boolean logic operators in conjunction with medical subject terms.

### Selection criteria

We first reviewed the titles and abstracts of all searched articles and then extracted all observational study indicators. All citations that met the criteria were evaluated, and those that didn't or were repeated were removed. Further evaluation of the article's relevance occurred by reading the full text carefully, evaluating references in the article, and reviewing relevant reviews to locate additional candidate studies. All references were classified and managed by Endnote X9 (Research Software, Philadelphia, United States).

### Inclusion and exclusion criteria

The inclusion criteria were as follows: (1) All patients in the included group were diagnosed with pituitary apoplexy through clinical manifestations and imaging examinations. (2) At least 5 patients were included in each trial. (3) The treatment regimen of the trial patients included conservative treatment and surgical treatment. (4) The main observation indicators were “visual acuity recovery”, “visual field recovery”, “ocular palsy recovery”, and “pituitary endocrine function”. (5) The patient's main observation data was obtained through continuous observation. The exclusion criteria were as follows: (1) The type of article is a case report, letter, or review. (2) The test subjects are not human (e.g., laboratory rats or other animals, etc.).

### The definition of the outcome

Visual recovery is defined as an improvement in a patient's vision following treatment, typically assessed using standard vision charts or logarithmic visual acuity charts. Visual field recovery refers to the improvement of visual field defects after treatment, which can be evaluated through computerized perimetry tests such as the Goldmann perimeter or Humphrey visual field analyzer. Ophthalmoplegia recovery denotes the resolution or improvement of ophthalmoplegia symptoms post-treatment, often involving dysfunction of the third, fourth, and sixth cranial nerves that lead to eye movement disorders. Pituitary function recovery reflects the normalization or improvement of pituitary hormone levels after treatment, with common manifestations including deficiencies in one or more hormones such as adrenocorticotropic hormone (ACTH), thyroid-stimulating hormone (TSH), growth hormone (GH), and gonadotropins (LH and FSH).

### Data extraction and quality assessment

For accuracy, two authors independently extracted data from eligible articles according to the inclusion and exclusion criteria and then discussed the results together, with any disagreements resolved by a third researcher. The baseline data of the included studies included the year and country of publication, the study design, the quality of the articles, and the fundamental characteristics of the experiments. We used Review Manager (Version 5.3) to evaluate the risk of bias in these studies. Additionally, we employed the Journal Citation Reports (JCR) classification to assess article quality, with quartiles Q1–Q4 representing grades I to IV, respectively. We employed the Cochrane Risk of Bias tool to assess the quality of the included studies (Supplementary Figures S4, S5).

### Statistical analysis

We recorded the number of patients undergoing surgical and conservative treatments both before and after surgery, focusing on the primary observations. The recovery rates for the four main observations were assessed using Reman v5.3. A fixed-effect model evaluated the pooled odds ratios (OR), where an OR of less than 1 favored surgical treatment, and an OR greater than 1 favored conservative management. We assessed the heterogeneity between studies using the Cochran Q (chi-square) test and *I*² statistics. A 95% confidence interval (CI) or a *p*-value of less than 0.05 was considered indicative of statistical significance for the included studies.

## Results

### Literature screening process and basic patient characteristics

**Figure 1:**
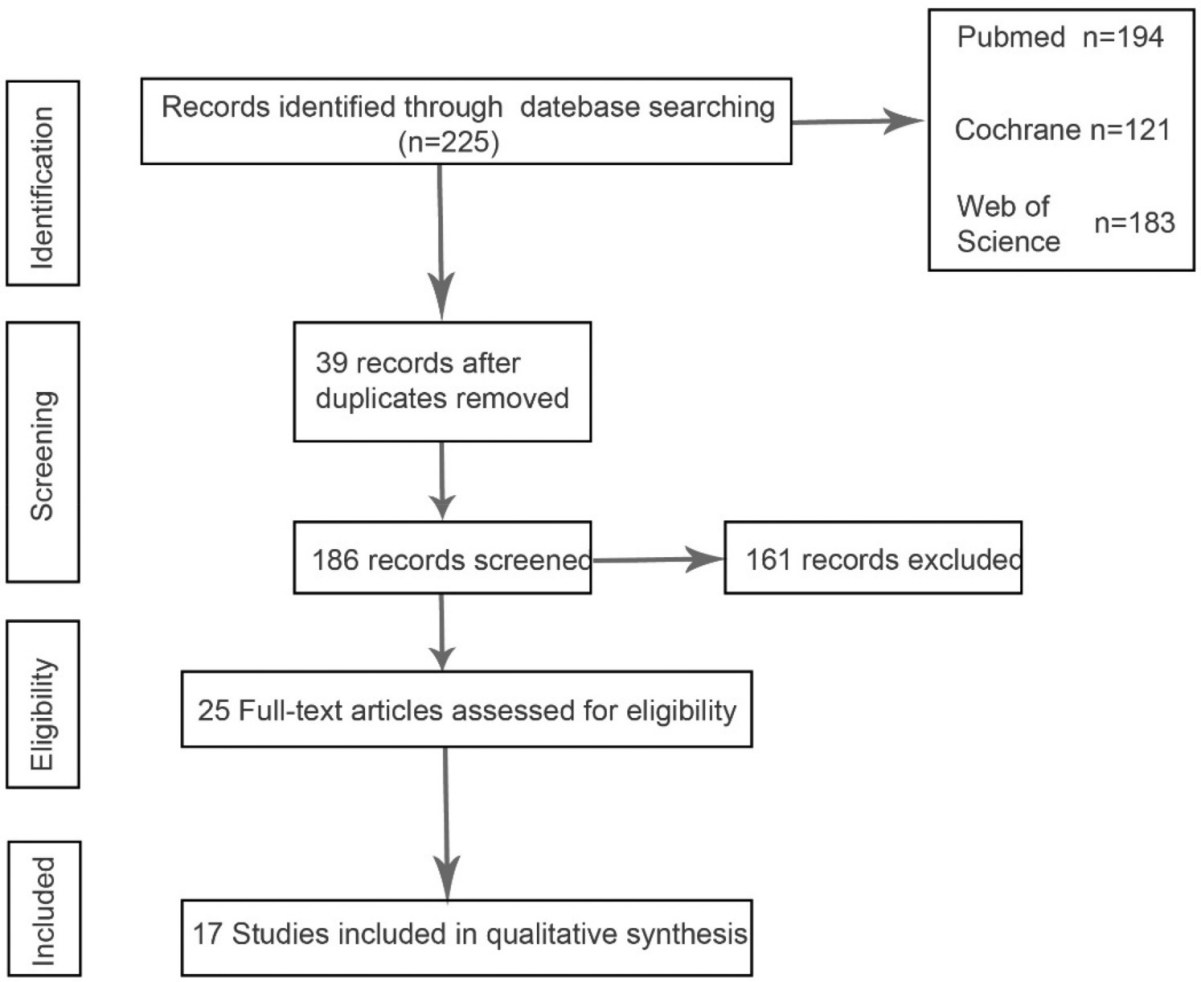
PRISMA flow chart.

Through keyword search, 225 articles were preliminarily screened, and after further screening of inclusion and exclusion criteria, 17 articles finally met the criteria. Figure 1 describes the process of article selection. The included studies were published between 1991 and 2024. Supplementary Figure S1 summarizes the basic characteristics of the included studies and the participating patients. The patients in the included literature were all diagnosed with pituitary apoplexy through clinical diagnosis and imaging examinations, of which 14 studies were retrospective studies and 3 studies were prospective studies. The patients included in the literature were all treated with surgery or conservative treatment, and the final reported observation indicators in the literature included one or more of the four items of “visual acuity recovery”, “visual field recovery”, “ocular palsy recovery”, and “pituitary endocrine function”. In Supplementary Figures S2, S3 we compared the changes in the number of patients before and after surgery or conservative treatment, and the number of patients with improved observation indicators after treatment is listed in the table.

### Details of surgical treatment and conservative treatment

The nasal approach and craniotomy are generally used for stroke surgery, with the nasal approach being the most used (9, 10). Conservative treatment generally includes glucocorticoid therapy, maintenance of water and electrolyte balance, and symptomatic supportive care. However, the choice between surgical treatment and conservative treatment is still a difficult problem in stroke treatment.

### Date-analysis of recovery rates for key observations

**Figure 2:**
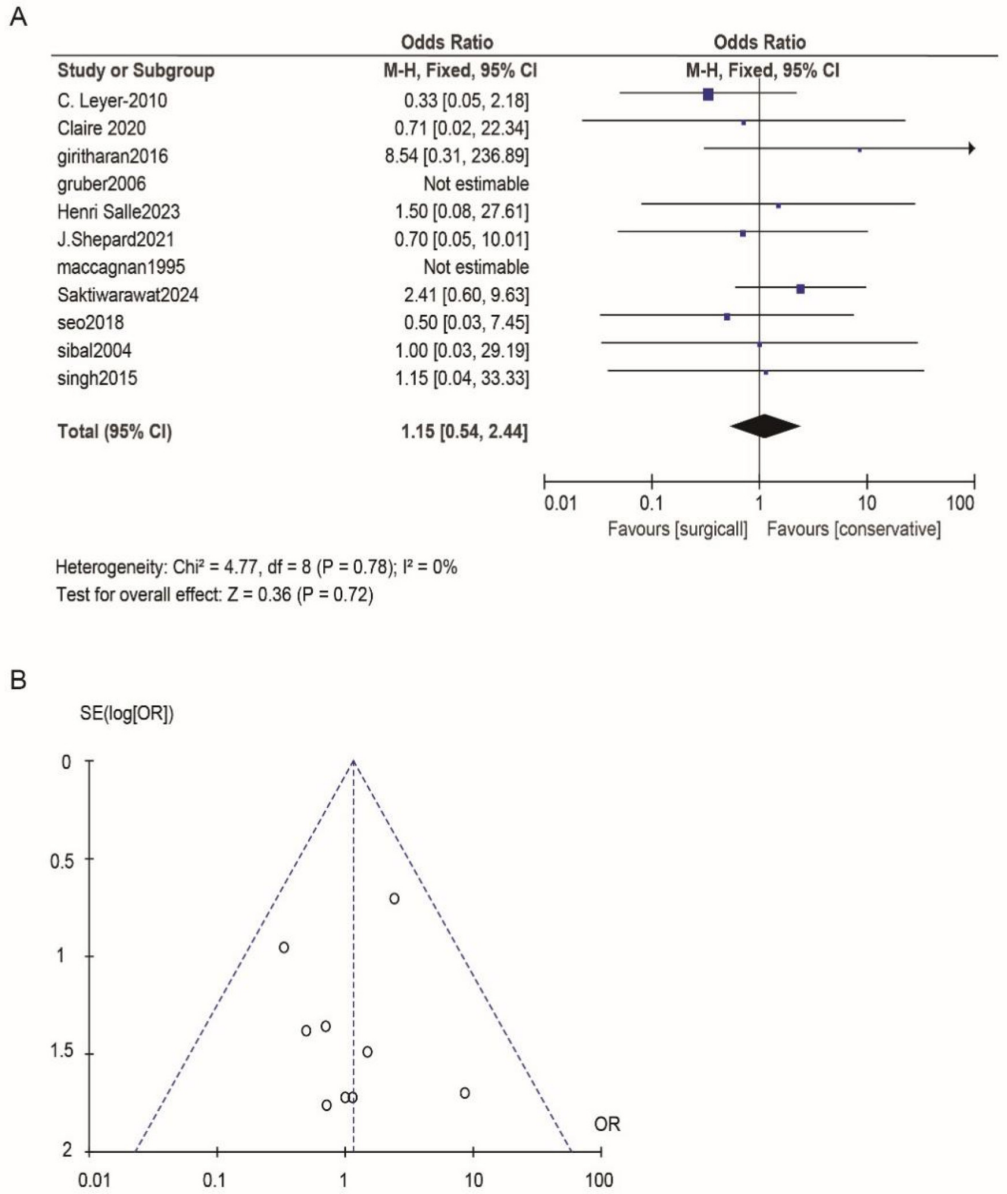
**(A)** Surgical versus conservative treatment efficacy aiming for visual acuity was compared. **(B)** Funnel plot for detecting and displaying system heterogeneity.

**Figure 3:**
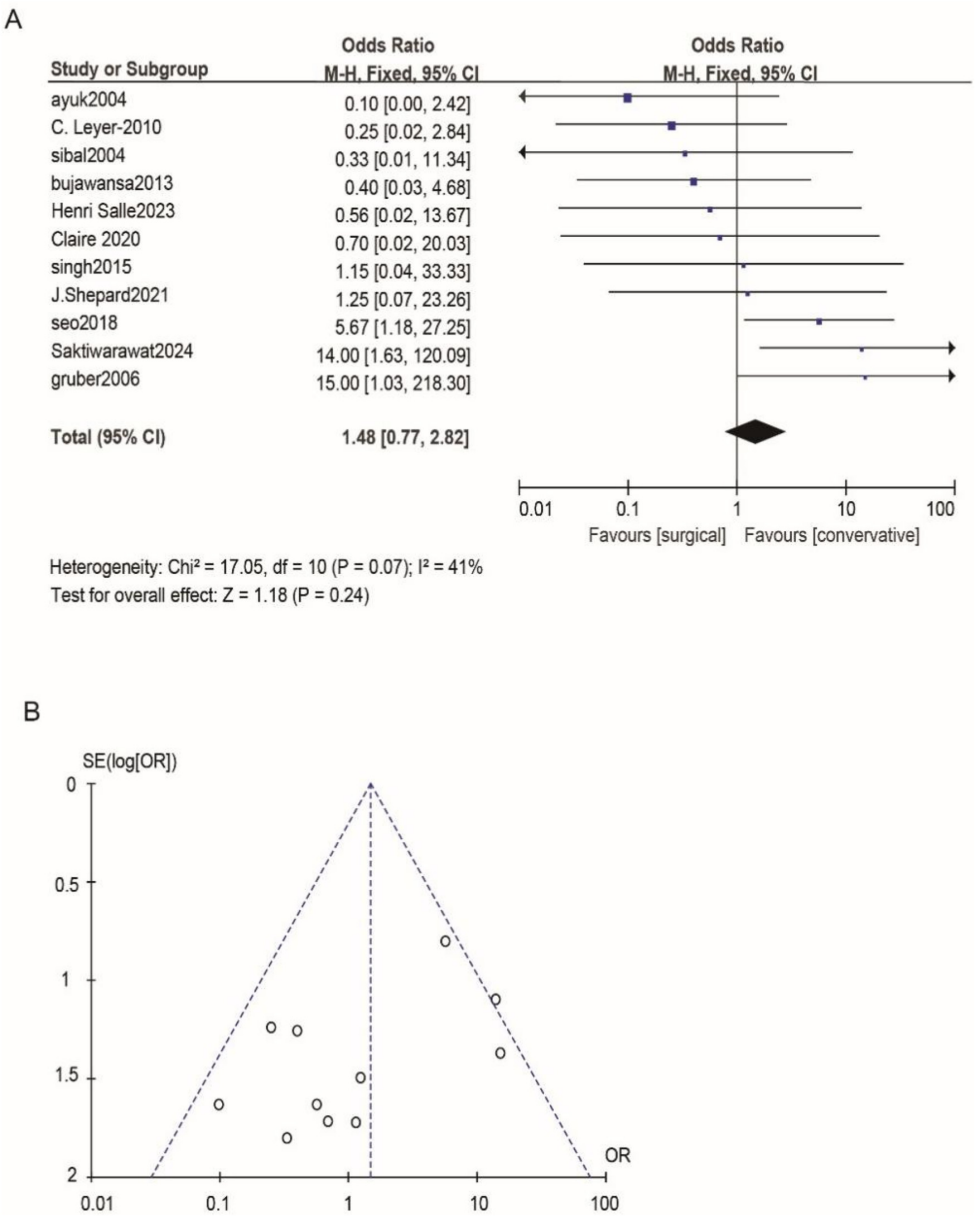
**(A)** The surgical treatment was compared to the conservative treatment for its efficacy in the visual field. **(B)** Funnel plot for detecting and displaying system heterogeneity.

**Figure 4:**
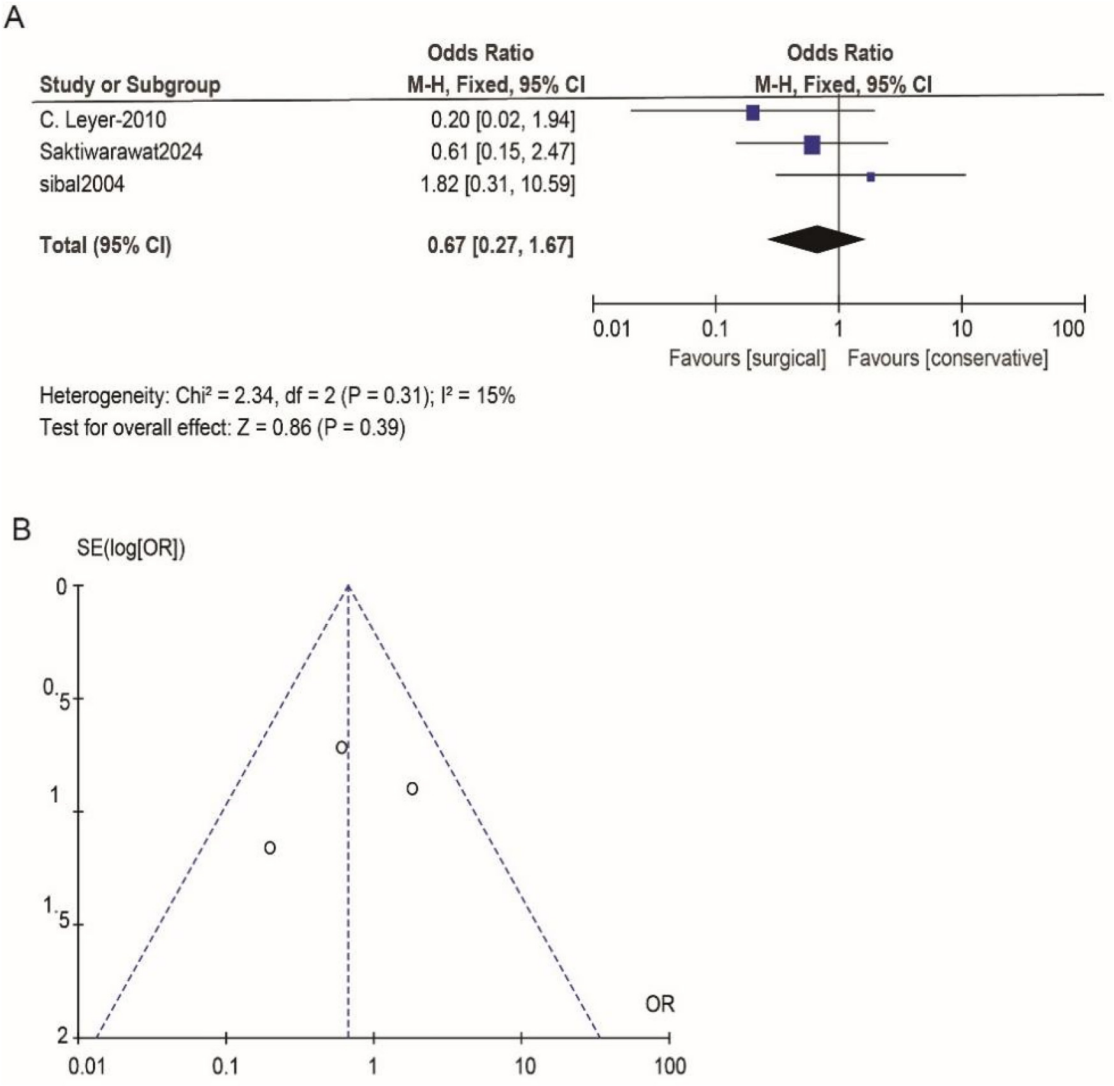
**(A)** Conservative and surgical treatments were compared in terms of their effectiveness in treating pituitary endocrine function. **(B)** Funnel plot for detecting and displaying system heterogeneity.

**Figure 5:**
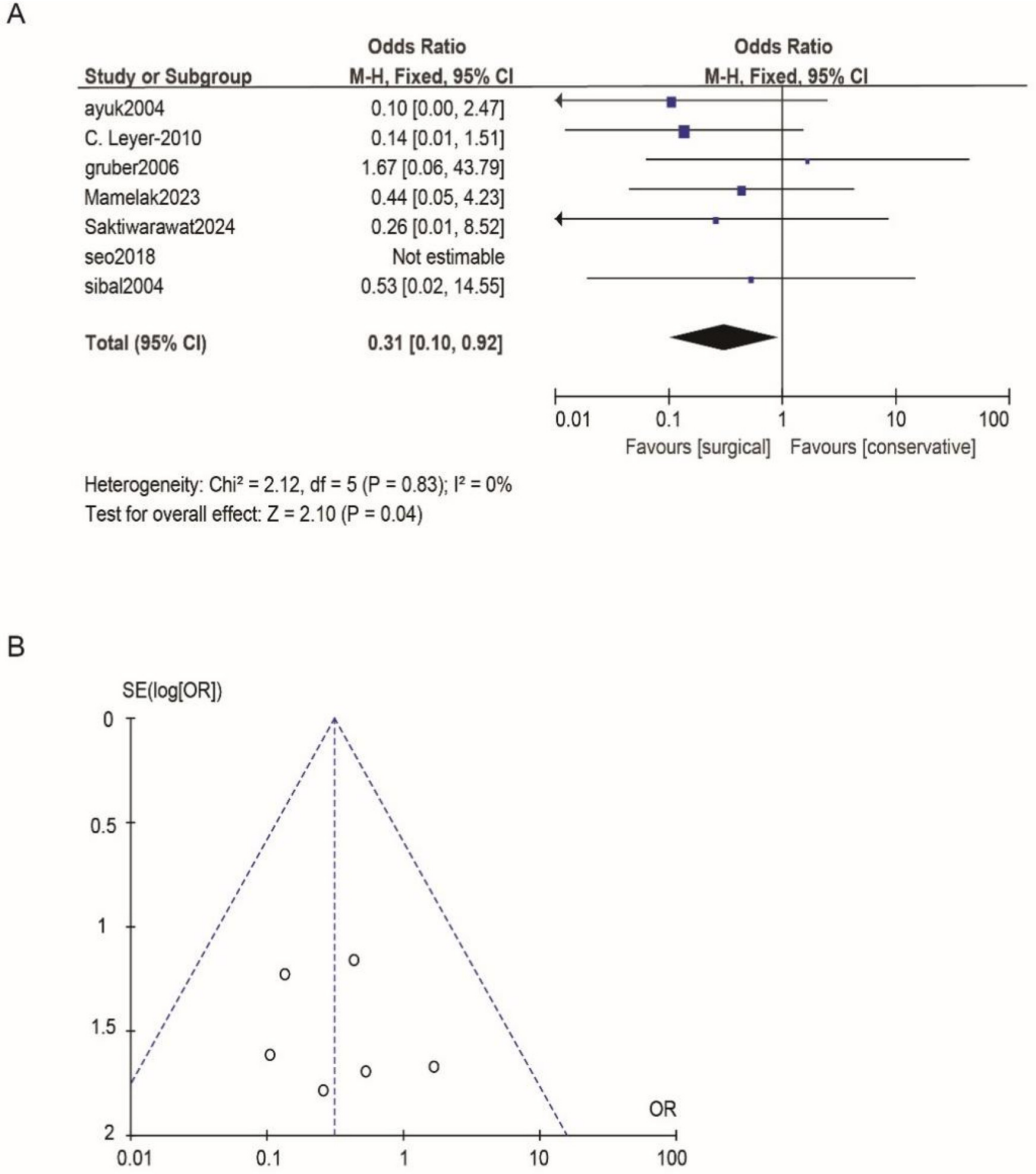
**(A)** Comparing the efficacy of conservative treatments and surgical treatments for ocular palsy. **(B)** Funnel plot for detecting and displaying system heterogeneity.

According to Reman v5.3 analysis, the comparison results of the main observed indicators after surgery or conservative treatment were as follows: surgical treatment compared with conservative treatment, postoperative visual acuity recovery (OR: 1.15; 95% CI 0.54–2.44; *p* = 0.72; Figure 2A), visual field recovery (OR: 1.48; 95% CI 0.77–2.82; *p* = 0.24; Figure 3A), and pituitary endocrine function (OR: 0.67; 95% CI). 0.27–1.67; *p* = 0.38; Figure 4A). However, there were significant differences in recovery from ocular palsy recovery (OR: 0.31; 95% CI 0.10–0.92; *p* = 0.04; Figure 5A) between surgical and conservative treatment groups. Heterogeneity in ophthalmoplegia recovery was low between studies (*I*^2^ = 0%, *p* = 0.83; Figure 5A). The inverted funnel plot shows that the scattering point is lower, indicating that the sample size may be on the low side (Figures 2–5B).

## Discussion

The challenge in treating pituitary apoplexy lies in deciding between conservative therapy and surgical intervention. Although many believe that surgery should be performed for pituitary apoplexy with significant neurological deficits, such as ophthalmological symptoms or a persistent decline in consciousness, there are no clear criteria. Moreover, some retrospective studies have shown no significant differences in the recovery of vision and endocrine function between patients treated conservatively and those treated with surgical decompression. Currently, there is a lack of high-level evidence-based medical evidence to guide the choice of treatment (11, 12). Consequently, we have summarized clinical data on conservative and surgical treatments for pituitary apoplexy over the past three decades, with the hope of providing a higher level of evidence-based medical evidence for the treatment of pituitary apoplexy, facilitating decision-making in clinical treatment plans.

Our study includes several literatures (8, 12–17) revealing that surgical intervention significantly improves ocular palsy compared to conservative measures, yet it does not substantially enhance vision acuity (12, 14–23), visual field (12–18, 20, 21, 23, 24), and pituitary function (12, 14, 16). Ocular muscle paralysis is likely attributable to transient ischemia, whereas visual and visual field impairments are typically associated with prolonged ischemia that induces neuronal necrosis and limits postoperative recovery. Moreover, visual and visual field deficits, along with endocrine dysfunction, often entail irreversible structural damage or chronic pathological changes, which may result in suboptimal postoperative outcomes. Postoperative treatment following pituitary apoplexy demonstrates limited significant improvements in visual and endocrine recovery. We attribute this to several factors: First, the inherent limitations of research design. Retrospective studies frequently lack standardized evaluation criteria, which introduces variability across studies. Additionally, differences among research centers, surgeons’ experience levels, timing of surgery, and surgical techniques may further influence postoperative recovery outcomes. Lastly, the shortcomings of outcome measurement indicators are notable. Visual assessments predominantly rely on the Snellen visual acuity chart, which has insufficient sensitivity for detecting specific visual field defects, such as bitemporal hemianopia. Endocrine evaluations often focus solely on hormone replacement requirements, potentially overlooking subclinical recoveries (e.g., positive ACHI stimulation tests despite normal basal cortisol levels). American scholars have also proposed a similar grading system for pituitary apoplexy: Grade 1—No significant symptoms, pituitary apoplexy is discovered incidentally; Grade 2—Manifest endocrine dysfunction; Grade 3—Headache; Grade 4—Ocular muscle paralysis; Grade 5—Acute vision loss or altered consciousness. They suggest that patients with Grades 1–3 should be treated conservatively, while those with Grades 4 and above should be considered for surgical treatment (25). The philosophy of this article aligns with our analysis, but we have summarized clinical data from nearly three decades, providing a higher level of evidence-based medicine for the conservative or surgical treatment of pituitary apoplexy, facilitating clinical decision-making for physicians. This study exhibits several limitations, primarily because the majority of studies failed to delineate the specific type of pituitary apoplexy, precluding subgroup analysis based on bleeding or ischemic characteristics (26, 27). Additionally, some low-quality data could potentially undermine the validity of our analysis.

Pituitary adenomas are among the most common intracranial tumors, and patients with pituitary apoplexy are also very common. These patients and their families often face physical and mental suffering as well as the burden of family finances. Therefore, we call for more research institutions to pay attention to the scientific research of pituitary apoplexy, in order to improve the level of clinical decision-making evidence and benefit patient prognosis.

## Conclusion

In summary, our findings suggest that surgical intervention is more effective than conservative approaches in improving ocular palsy recovery rates following pituitary apoplexy, and these results demonstrate evidence-based medical support for guiding clinical decision-making. However, the findings of this meta-analysis are constrained by the limitations of the current studies. Future research should focus on conducting more randomized controlled trials and acquiring higher-quality data to further validate and support these conclusions.

## Data Availability

The original contributions presented in the study are included in the article/Supplementary Material, further inquiries can be directed to the corresponding authors.

## Supplementary material

Supplementary Figure S1 Characteristics of included studies.

Supplementary Figure S2 Recovery rates of visual acuity and pituitary function deficiency included in the study.

Supplementary Figure S3 Recovery rates of the visual field and Ocular palsy included in the study.

Supplementary Figure S4 Risk of bias graph: the judgments about each risk of bias item are presented as percentages across all included studies.

Supplementary Figure S5 Risk of bias summary: the judgments about each risk of bias item for each included study.
