# Supplementary figures and images for: Pituitary apoplexy: surgical or conservative? A meta-analytical insight
Source: Front Surg. 2025 Jun 4;12:1579498. doi: 10.3389/fsurg.2025.1579498 (PMC12174462; doi:10.3389/fsurg.2025.1579498)

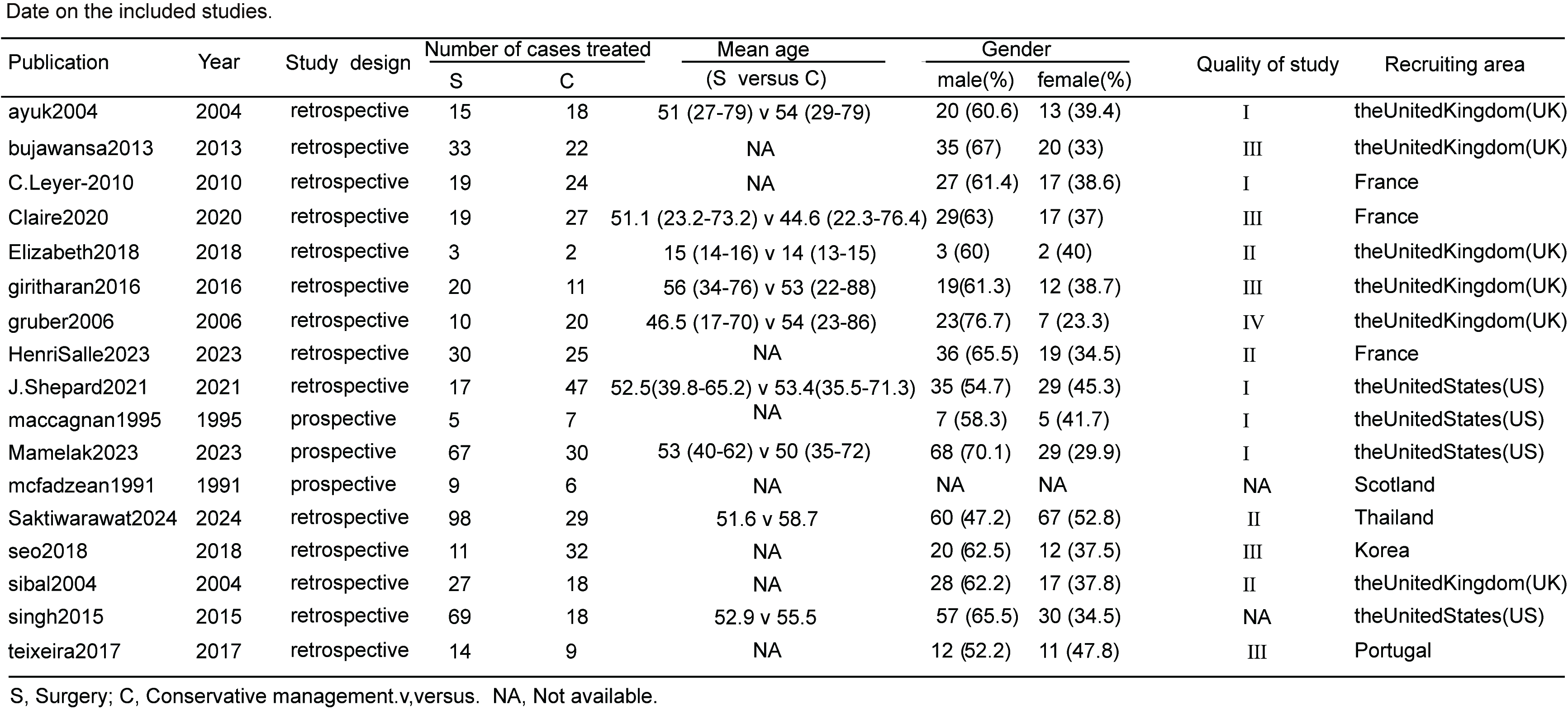

Supplement: Supplementary Figure S1 — Characteristics of included studies. [file Image1.tif]

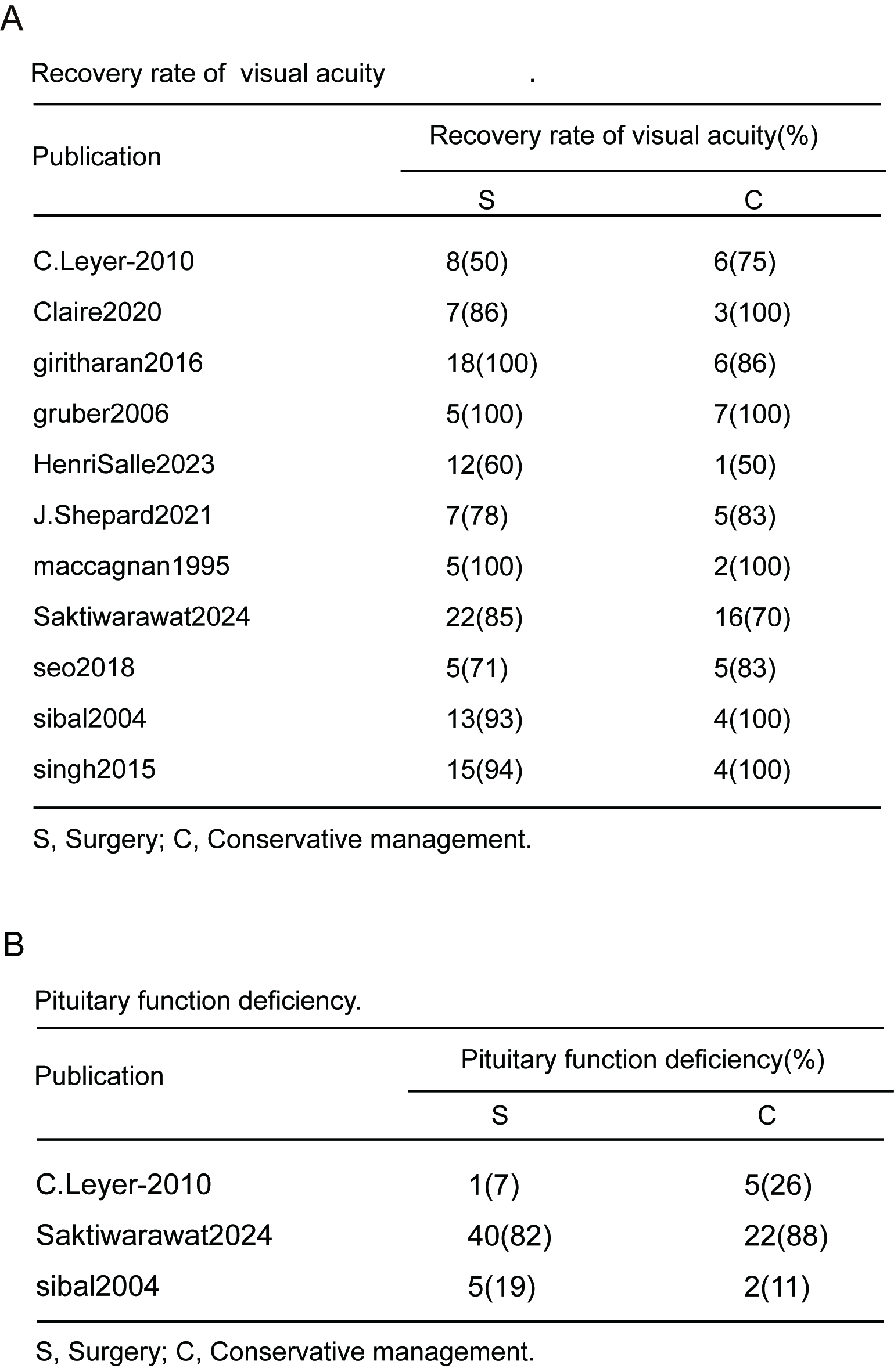

Supplement: Supplementary Figure S2 — Recovery rates of visual acuity and pituitary function deficiency included in the study. [file Image2.tif]

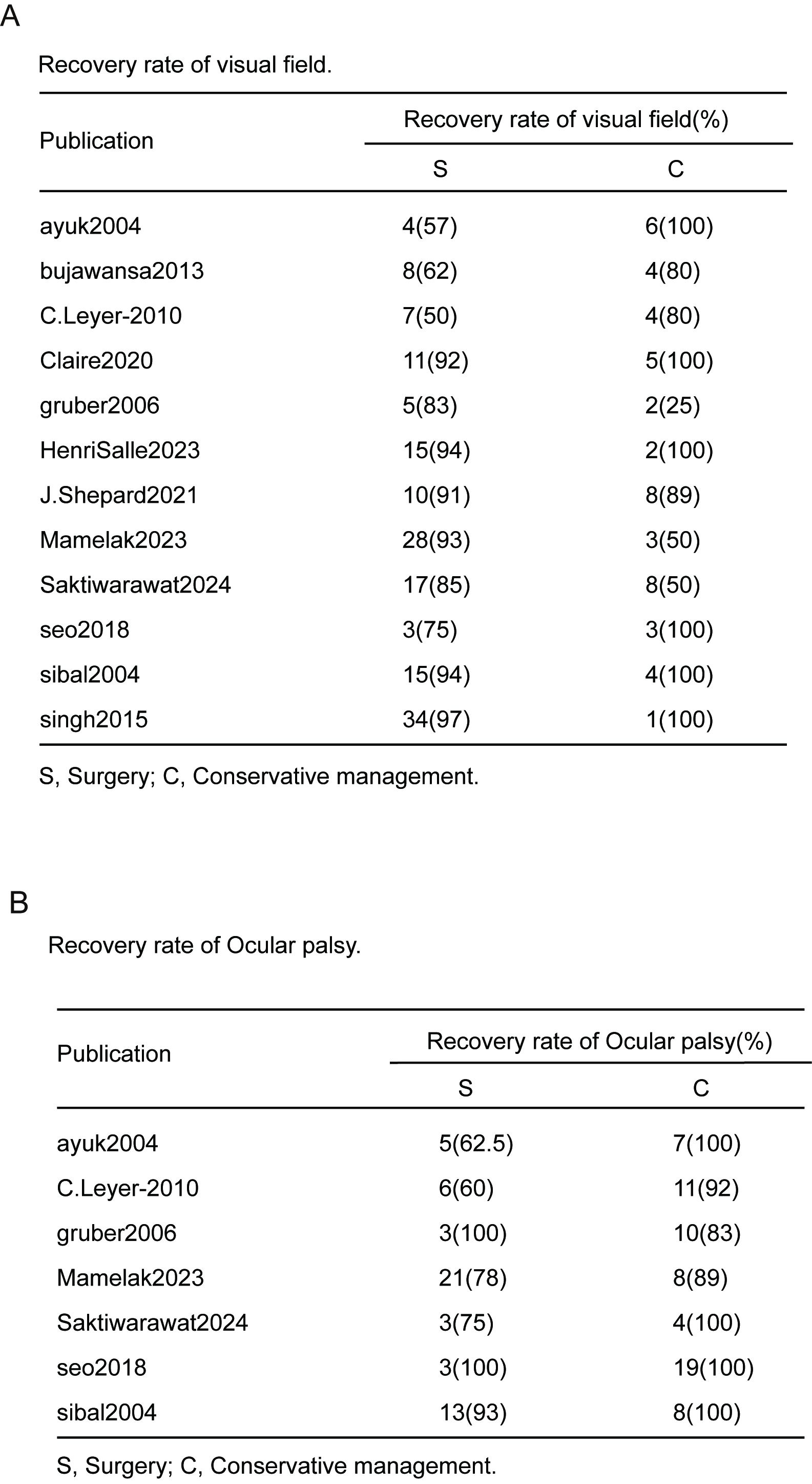

Supplement: Supplementary Figure S3 — Recovery rates of the visual field and Ocular palsy included in the study. [file Image3.tif]

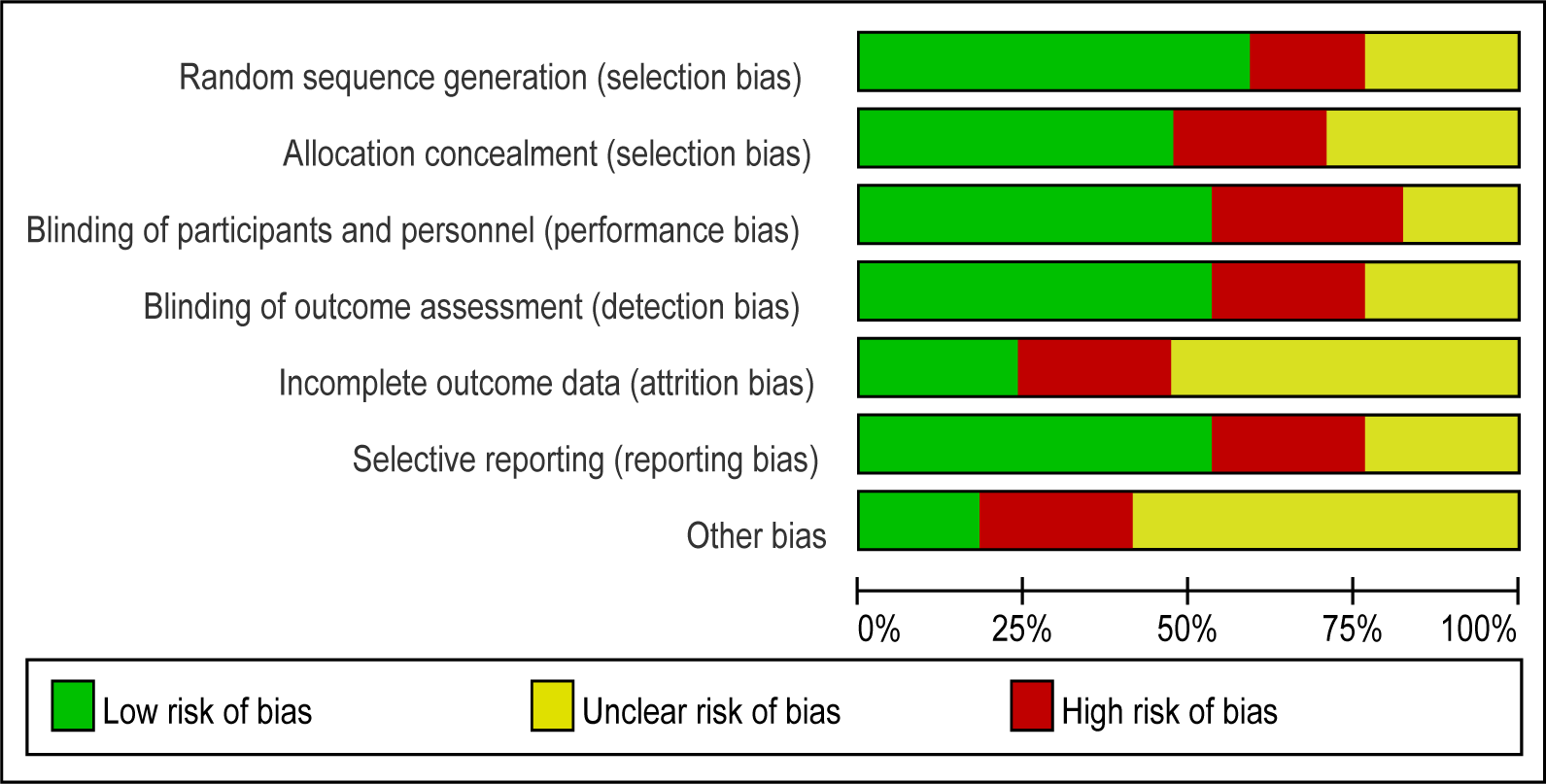

Supplement: Supplementary Figure S4 — Risk of bias graph: the judgments about each risk of bias item are presented as percentages across all included studies. [file Image4.tif]

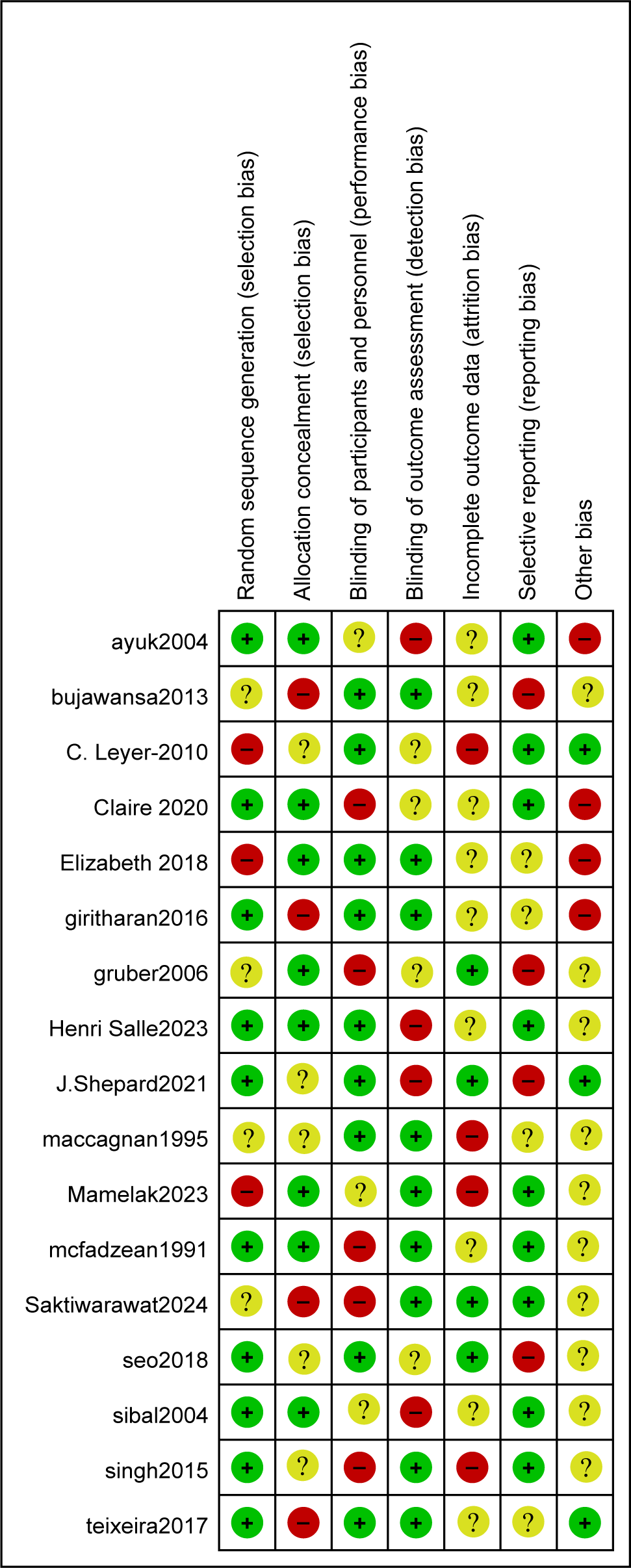

Supplement: Supplementary Figure S5 — Risk of bias summary: the judgments about each risk of bias item for each included study. [file Image5.tif]
